# Supplementary material for: A BALB/c IGHV Reference Set, Defined by Haplotype Analysis of Long-Read VDJ-C Sequences From F1 (BALB/c x C57BL/6) Mice
Source: Front Immunol. 2022 Jun 3;13:888555. doi: 10.3389/fimmu.2022.888555 (PMC9205180; doi:10.3389/fimmu.2022.888555)
Supplement: Supplementary file 1 [file Table_1.pdf]

Supplementary Table I: Mouse IGHV sequences in the IMGT Reference Directory that have been changed since the analysis by Collins *et al* (Collins et al., 2015), with altered or added sequences in uppercase.

IGHV2-9*02<br><br>(previously named IGHV2-9-1*01)	caggtgcagctgaaggagtcaggacctggcctggtggcgccctcacagagcctgtccatcacttgcactgtctctgggttttcattaaccagctatggtgtacactgggttcgccagcctccaggaaagggtctggagtggctgggagtaatatgggctggtggaagcacaaattataattcggctctcatgtccagactgagcatcagcaaagacaactccaagagccaagttttcttaaaaatgaacagtctgcaaactgatgacacagccatgtactactgtgccagaga
IGHV2-9-1*01<br><br>(previously musIGHV211)	caggtgcagctgaaggagtcaggacctggcctggtggcgccctcacagagcctgtccatcacatgcactgtctctgggttctcattaaccagctatgctataagctgggttcgccagccaccaggaaagggtctggagtggcttggagtaatatggactggtggaggcacaaattataattcagctctcaaatccagactgagcatcagcaaagacaactccaagagtcaagttttcttaaaaatgaacagtctgcaaactgatgacacagccaggtactactgtgccagaAA
IGHV2-9-2*01<br><br>(previously IGHV2-9*02)	caggtgcaactgaaggagtcaggacctggcctggtggcgccctcacagagcctgtccattacctgcactgtctctgggttctcattaaccagctatgatataagctggattcgccagccaccaggaaagggtctggagtggcttggagtaatatggactggtggaggcacaaattataattcagctttcatgtccagactgagcatcagcaaggacaactccaagagccaagttttcttaaaaatgaacagtctgcaaactgatgacacagccatatattactgtgtaagaga
IGHV4-1*01	gaggtgaagcttctccagtctggaggtggcctggtgcagcctggaggatccctgaaactctcctgtgcagcctcaggaatcgattttagtagatactggatgagttgggttcggcgggctccagggaaaggactagaatggattggagaaattaatccagatagcagtacaataaactatgcaccatctctaaaggataaattcatcatctccagagacaacgccaaaaatacgctgtacctgcaaatgagcaaagtgagatctgaggacacagccctttattactgtgcaagaCC
IGHV5-6-5*01<br><br>(previously IGHV5S12*01)	gaagtgaagctggtggagtctgggggaggcttagtgaagcctggagggtccctgaaactctcctgtgcagcctctggattcactttcagtagctatgccatgtcttgggttcgccagactccagagaagaggctggagtgggtcgcatccattagtagtggtggtagcacctactatccagacagtgtgaagggccgattcaccatctccagagataatgccaggaacatcctgtacctgcaaatgagcagtctgaggtctgaggacacggccatgtattactgtgcaagagg
IGHV5-15*01	gaggtgaagctggtggagtctgggggaggcttagtgcagcctggagggtccctgaaactctcctgtgcagcctctggattcactttcagtgactacggaatggcgtgggttcgacaggctccaaggaaggggcctgagtgggtagcattcattagtaatttggcatatagtatctactatgcagacactgtgacgggccgattcaccatctctagagagaatgccaagaacaccctgtacctggaaatgagcagtctgaggtctgaggacacggccatgtattactgtgcaagaCA
IGHV5-16*01	gaagtgaagctggtggagtctgagggaggcttagtgcagcctggaagttccatgaaactctcctgcacagcctctggattcactttcagtgactattacatggcttgggtccgccaggttccagaaaagggtctagaatgggttgcaaacattaattatgatggtagtagcacctactatctggactccttgaagagccgtttcatcatctcgagagacaatgcaaagaacattctatacctgcaaatgagcagtctgaagtctgaggacacagccacgtattactgtgcaagaGA
IGHV7-1*01	gaggtgaagctggtggaatctggaggaggcttggtacagtctgggcgttctctgagactctcctgtgcaacttctgggttcaccttcagtgatttctacatggagtgggtccgccaagctccagggaagggactggagtggattgctgcaagtagaaacaaagctaatgattatacaacagagtacagtgcatctgtgaagggtcggttcatcgtctccagagacacttcccaaagcatcctctaccttcagatgaatgccctgagagctgaggacactgccatttattactgtgcaagaGATGCA

IGHV8-6*01	caggttactctgaaagagtctggccctggtatattgcagccctcgcagaccctcagtctgacttgttctttctctgggttttcactgagtacttttggtatgggtgtgagctggattcgtcagccttcagggaaggatctggagtggctggcacacatttattgggatgatgacaagcactataacccatccttgaagagccagctcagaatctccaaggatacctccaacaaccaggtattcctcaagatcaccactgtggacactgtagatactgccacatactactgtgctcgaAGAG
IGHV10-3*01	gaggtgcagcttgttgagtctggtggaggattggtgcagcctaaaggatcattgaaactctcatgtgccgcctctggtttcaccttcaatacctatgccatgcactgggtccgccaggctccaggaaagggtttggaatgggttgctcgcataagaagtaaaagtagtaattatgcaacatattatgccgattcagtgaaagacagattcaccatctccagagatgattcacaaagcatgctctatctgcaaatgaacaacctgaaaactgaggacacagccatgtattactgtgtgagagA
