# Supplementary material for: A BALB/c IGHV Reference Set, Defined by Haplotype Analysis of Long-Read VDJ-C Sequences From F1 (BALB/c x C57BL/6) Mice
Source: Front Immunol. 2022 Jun 3;13:888555. doi: 10.3389/fimmu.2022.888555 (PMC9205180; doi:10.3389/fimmu.2022.888555)
Supplement: Supplementary file 2 [file Table_2.pdf]

Supplementary Table II: Extended sequences of apparently truncated IGHV sequences previously reported as being from the BALB/c or related strains.

Gene Name	Full Length Sequence*
IGHV1S113*01	gaggtccagctgcaacagtctggacctgagctggtgaagcctggggcttcagtgaagatatcctgcaagacttctggatacacattcactgaatacaccatgcactgggtgaagcagagccatggaaagagccttgagtggattggaggtattaatcctaacaatggtggtactagctacaaccagaagttcaagggcaaggccacattgactgtagacaagtcctccagcacagcctacatggagctccgcAGCCTGACATCTGAGGATTCTGCAGTCTATTACTGTGCAAGA
IGHV1S122*01	caggtccaactccagcagcctggggctgaactggtgaagcctggggcttcagtgaagttgtcctgcaaggcttctggctacaccttcaccagctactggatgcactgggtgaagctgaggcctggacaaggctttgagtggattggagagattaatcctagcaatggtggtactaactacaatgagaagttcaagagaaaggccacactgactgtagacaaatcctccagcacagcctacatgcaactcagcAGCCTGACATCTGAGGACTCTGCGGTCTATTACTGTACAATA
IGHV1S22*01	CAGGTCCAActgcagcaacctgggtctgagctggtgaggcctggagcttcagtgaagctgtcctgcaaggcttctggctacacattcaccagctactggatgcactgggtgaagcagaggcatggacaaggccttgagtggattggaaatatttatcctggtagtggtagtactaactacgatgagaagttcaagagcaagggcacactgactgtagacacatcctccagcacagcctacatgcacctcagcagcctgacatctgaggactctgcggtctattactgtacaaga
IGHV1S33*01	CAGGTTCAGctgcagcagtctggacctgagctggtgaagcctggggctttagtgaagatatcctgcaaggcttctggttacaccttcacaagctacgatataaactgggtgaagcagaggcctggacagggacttgagtggattggatggatttatcctggagatggtagtactaagtacaatgagaaattcaagggcaaggccacactgactgcagacaaatcctccagcacagcctacatgcagctcagcagcctgacttctgagaactctgcagTCTATTTCTGTGCAAGA
IGHV1S34*01	GAGGTCCAGCTGCAGCAGTCTGGACCTGAGCTAGTGAAGACTGGGGCTTCAGTGAAGatatcctgcaaggcttctggttactcattcactggttactacatgcactgggtcaagcagagccatggaaagagccttgagtggattggatatattagttgttacaatggtgctactagctacaaccagaagttcaagggcaaggccacatttactgtagacacatcctccagcacagcctacatgcagttcaacagcctgacatctgaagactctgcggtctattactgtgcaaga
IGHV1S68*02	caggtccaactgcagcagcctggggctgagcttgtgaagcctgggacttcagtgaagctgtcctgcaaggcttctggctacaacttcaccagctactggataaactgggtgaagctgaggcctggacaaggccttgagtggattggagatatttatcctggtagtggtagtactaactacaatgagaagttcaagagcaaggccacactgactgtagacacatcctccagcacagcctacatgcaactcagcAGCCTGGCATCTGAGGACTCTGCTCTCTATTACTGTGCAAGA
IGHV1S72*01	caggtccaactgcagcagcctggggctgagcttgtgaagcctggggctccagtgaagctgtcctgcaaggcttctggctacaccttcaccagctactggatgaactgggtgaagcagaggcctggacgaggcctcgagtggattggaaggattgatccttccgatagtgaaactcactacaatcaaaagttcaaggacaaggccacactgactgtagacaaatcctccagcacagcctacatccaactcagcAGCCTGACATCTGAGGACTCTGCGGTCTATTACTGTGCAAGA

IGHV1S75*01<br><br>**	caggtccaactgcagcagcctggggctgagcttgtgaagcctggggcttcagtgaaaatgtcctgcaag<br>gcttctggctacaccttcaccagctactggataaactgggtgaagcagaggcctggacaaggccttgagt<br>ggattggagatatttatcctggtagaggtattactaactacaatgagaagttcaagagcaaggccacactg<br>actctagacacatcctccagcacagcctacatgcagctcagcAGCCTGACATCTGAGGA<br>CTCTGCGGTCTATTATTGTTCAAGA
IGHV1S82*01<br><br>***	caggtccaactgcagcagcctggggctgagctggtgaggcctggagcttcagtgaagctgtcctgcaag<br>gcttctggctactccttcaccagctactggatgaactgggtgaagcagaggcctggacaaggccttgagt<br>ggattggcatgattcatccttccgatagtgaaactaggttaaatcagaagttcaaggacaaggccacattg<br>actgtagacaaatcctccagcacagcctacatgcaactcagcAGCCCGACATCTGAGGA<br>CTCTGCGGTCTATTACTGTGCAAGA
IGHV9S8*01<br><br>****	cagatccagttggtgcagtctggacctgagctgaagaagcctggagagacagtcaagatctcctgcaag<br>gcttctgggtataccttcacaaactatggaatgaactgggtgaagcaggctccaggaaagggtttaaagtg<br>gatgggctggataaacactgagactggtgagccaacatatgcagatgacttcaagggacggtttgccttct<br>ctttggaaacctctgccagcactgcctatttgcagatcaacaacctcaaaaatgaggacacggctacatatt<br>tctgtGCAAGA
balbIGHV023	caggttactctgaaagagtctggccctgggatattgcagccctcccagaccctcagtctgacttgttctttct<br>ctgggttttcactgagcacttctggtatgggtgtgagctggattcgtcagccttcaggaaagggtctggagt<br>ggctggcacacatttactgggatgatgacaagcgctataacccatccctgaagagccggctcacaatctc<br>caaggatacctccagaaaccaggtattcctcaagatcaccagtgtggacactgcagatactgccacatac<br>tactgtgctcgaAGAG
balbIGHV026	caggttactctgaaagagtctggccctgggatattgcagccctcccagaccctcagtctgacttgttctttct<br>ctgggttttcactgagcacttctggtatgggtgtaggctggattcgtcagccttcagggaagggtctggagt<br>ggctggcacacatttggtgggatgatgacaagcgctataacccagccctgaagagccgactgacaatct<br>ccaaggatacctccagcaaccaggtattcctcaagatcgccagtgtggacactgcagatactgccacata<br>ctactgtgctcgaataG
balbIGHV028	caggttactctgaaagagtctggccctgggatattgcagccctcccagaccctcagtctgacttgttctttct<br>ctgggttttcactgagcacttctggtatgagtgtaggctggattcgtcagccttcagggaagggtctggagt<br>ggctggcacacatttggtggaatgatgataagtactataacccagccctgaaaagccggctcacaatctc<br>caaggatacctccaacaaccaggtattcctcaagatcgccagtgtggtcactgcagatactgccacatact<br>actgtgctcgaataG
balbIGHV030	caggttactctgaaagagtctggccctgggatattgcagccctcccagaccctcagtctgacttgttctttct<br>ctgggttttcactgagcacttctggtatgggtgtaggctggattcgtcagccttcaggagagggtctagagt<br>ggctggcagacatttggtgggatgacaataagtactataacccatccctgaagagccggctcacaatctc<br>caaggatacctccagcaaccaggtattcctcaagatcaccagtgtggacactgcagatactgccacttact<br>actgtgctcgaagaG
balbIGHV032	caggtccagctgcagcagtctgggcctgagctggtgaggcctggggtctcagtgaagatttcctgcaag<br>ggttccggctacacattcactgattatgctatgcactgggtgaagcagagtcatgcaaagagtctagagtg<br>gattggagttattagtacttactctggtaatacaaactacaaccagaagtttaagggcaaggccacaatgac<br>tgtagacaaatcctccagcacagcctatatggaacttgccagattgacatctgaggattctgccatctattac<br>tgtGCAAGA
balbIGHV034	Caggttactctgaaagagtctggccctgggatattgcagccctcccagaccctcagtctgacttgttctttct<br>ctgggttttcactgagcacttctggtatgggtgtaggctggattcgtcagccttcagggaagggtctggagt<br>ggctggcacacatttggtgggatgatgataagtactataacacagccctgaagagcgggctcacaatctc

	caaggatacctccaaaaaccaggtcttcctcaagatcgccagtgtggacactgcagatactgccacatac<br>tactgtgctcgaataG
J558.27	caggtccagctgcagcagtctggacctgagctggtgaagcctggggcttcagtgaagatgtcctgcaag<br>gcttctggctacaccttcacaagctactatatacactgggtgaagcagaggcctggacagggacttgagt<br>ggattggatggatttatcctggagatggtagtactaagtacaatgagaagttcaagggcaagaccacactg<br>actgcagacaaatcctccagcacagcctacatgttgctcagcagcctgacctctgaggactctgcgatcta<br>tttctgtgcaagA
J558.44	caggtccagctgcagcagtctggggctgaactggcaagacctggggcctcagtgaagatgtcctgcaa<br>ggcttctggctacacctttactagctacacgatgcactgggtaaaacagaggcctggacagggtctggaa<br>tggattggatacattaatcctagcagtggttatactaattacaatcagaagttcaaggacaaggccacattg<br>actgcagacaaatcctccagcacagcctacatgcaactgagcagcctgacatctgaggactctgcagtct<br>attactgtgcaaGA
musIGHV021	caggttcagctccagcagtctggggctgagctggcaagacctggggcttcagtgaagttgtcctgcaag<br>gcttctggctacacctttactagctactggatgcagtgggtaaaacagaggcctggacagggtctggaat<br>ggattggggctatttatcctggagatggtgatactaggtacactcagaagttcaagggcaaggccacattg<br>actgcagataaatcctccagcacagcctacatgcaactcagcagcttggcatctgaggactctgcggtcta<br>ttactgtGCAAGA
musIGHV616	caggttactctgaaagagtctggccctgggatattgcagccctcccagaccctcagtctgacttgttctttct<br>ctgggttttcactgagcacttctaatatgggtgtaggctggattcgtcagccttcagggaagggtctggagt<br>ggctgttacacattttgtggaatgatagtaagtactataacccagccctgaagagccggctcacaatctcca<br>aggatacctacaacaaccaggtattcctcaagatcgccaatgtggacactgcagatactgccacatactac<br>tgtgctcgaATAG
musIGHV672	caggttattctgaaagagtctggccctggaatattgcagccctctcagaccctcagtctgacttgttctttctc<br>tgggttttcacttagcacttatggtacagctgtgaactggattcgtcagccttcaggaaagggtctggagtg<br>gttggcacaaattgggtcagatgatagcaagctctataacccatttctgaaaagccgaatcacaatctccaa<br>ggatacctccaacagccaggtattcctcaagatcactagtgtggacactgaagattctgccacatactact<br>gtgctaacAGA

* Extensions are shown in Uppercase
** Not confirmed in this study
*** The extension originally ended in the terminal nucleotides TA, but were determined to be GA by analysis of the rearranged gene ends.
**** Unable to be confirmed by haplotype analysis
