# Supplementary material for: A BALB/c IGHV Reference Set, Defined by Haplotype Analysis of Long-Read VDJ-C Sequences From F1 (BALB/c x C57BL/6) Mice
Source: Front Immunol. 2022 Jun 3;13:888555. doi: 10.3389/fimmu.2022.888555 (PMC9205180; doi:10.3389/fimmu.2022.888555)
Supplement: Supplementary file 4 [file Table_4.pdf]

Supplementary Table IV: Constant Region Genes inferred from VDJ-C datasets.

Label	Exon	Strain	Source
IGHG2A*01	CH1	BALB/c	IMGT
IGHG2A*01	Hinge	BALB/c	IMGT
IGHG2A*01	CH2	BALB/c	IMGT
IGHG2A*01	CH3-CHS	BALB/c	IMGT
IGHG2B*rs45969375c	CH1	BALB/c	Inferred
IGHG2B*03 (rs45969375t)	CH1	C57BL/6	IMGT[a]
IGHG2B*02/03 (identical)	Hinge	Both	IMGT
IGHG2B*02	CH2	BALB/c	IMGT
IGHG2B*03	CH2	C57BL/6	IMGT[a]
IGHG2C	CH1	C57BL/6	IMGT
IGHG2C*01	Hinge	C57BL/6	IMGT
IGHG2C*01	CH2	C57BL/6	IMGT
IGHG2C*03	CH3-CHS	C57BL/6	IMGT[b]
IGHM*rs29176517g	CH1	BALB/c	Inferred
IGHM*04 (rs29176517a)	CH1	C57BL/6	IMGT[a]
IGHM*02	CH2	Both	IMGT
IGHM*02	CH3	Both	IMGT

[a]The sequence can be found in the IMGT database, but is not present in the IMGT Mouse Gene Table. Its association with the strain was confirmed in this study using IGHJ1-based haplotyping of VDJ-C data.

[b]The sequence is available in the IMGT Mouse Gene Table, but is incorrectly assigned. Its association with the strain was confirmed by alignment with the Mouse Genome Reference Sequence.
