# Supplementary material for: A BALB/c IGHV Reference Set, Defined by Haplotype Analysis of Long-Read VDJ-C Sequences From F1 (BALB/c x C57BL/6) Mice
Source: Front Immunol. 2022 Jun 3;13:888555. doi: 10.3389/fimmu.2022.888555 (PMC9205180; doi:10.3389/fimmu.2022.888555)
Supplement: Supplementary file 5 [file Table_5.pdf]

Supplementary Table V: IGHV genes identified in the F1 mouse genotype that were unable to be haplotyped.

	Present in the mouse GRCm39/mm39 genome reference sequence	Present in the BALB_cJ_v1' sequence of the Mouse Genome Project (MGP)
IGHV1-14*01	Y	N
IGHV1-70*01	Y	N
IGHV1-79*01	Y	N
IGHV2-6-6*01	N	Y
IGHV3-3*02	N	Y
IGHV3-4*02	N	Y
IGHV5-1*02	N	Y
IGHV6-4*01	Y	N
IGHV6-4*02	N	N
IGHV1-34*01	Y	N
IGHV1-37*01	Y	N
IGHV8-4*01	Y	N
IGHV004b*01	N	N
IGHV014b*01	N	N
IGHV017b*01	N	Y
IGHV1S132*01	N	Y
IGHV9S8*01	N	N
balbIGHV039	N	N
